# PLACE-BASED HEALTH DISPARITIES: FUNCTIONAL DISABILITY IN APPALACHIAN WEST VIRGINIA

**DOI:** 10.1093/geroni/igac059.1856

**Published:** 2022-12-20

**Authors:** Carly Pullen, Kareem Ibrahim-Bacha, Julie Hicks Patrick

**Affiliations:** West Virginia University, Morgantown, West Virginia, United States; West Virginia University, Morgantown, West Virginia, United States; West Virginia University, Morgantown, West Virginia, United States

## Abstract

Place-based health disparities contribute to disability across the lifespan. Additional examinations of contributors to morbidity and disability at mid-and late-life are needed to inform policies and programs. Using data from the 2020 Centers for Disease Control and Prevention’s (CDC) Behavioral Risk Factor Surveillance System (BRFSS), we examine some of the social determinants of health (e.g., age, gender, education, income) as predictors of access to health care and predictors of functional ability. The study included 5,880 adults living in West Virginia all between the ages of 18 to 65+ years. Access to healthcare was indexed by three variables, including whether one had medical insurance, saw personal physicians, and avoided medical treatment due to cost. Functional ability was indexed by reports of difficulty in three ADLs and the number of chronic health conditions. Although most paths were significant, our initial model fit the data poorly, X2 (DF = 39, N =5880) = 2503.6, p < .001, CFI = .70, RMSEA = 1.0. We re-ran the model, using age as a moderator. In this set of analyses, the model fit well for middle-aged and older adults, but a different set of predictors characterized the relations for younger adults. Our results suggest that policies and programs that increase medical access for current middle-aged and older adults might decrease functional disability. Moreover, as younger adults age into midlife, they enter with lower economic and educational resources, further exacerbating their lack of access to health care and increasing disability.

